# Defective response of CD4^+ ^T cells to retinoic acid and TGFβ in systemic lupus erythematosus

**DOI:** 10.1186/ar3387

**Published:** 2011-06-27

**Authors:** Eric S Sobel, Todd M Brusko, Ed J Butfiloski, Wei Hou, Shiwu Li, Carla M Cuda, Ariana N Abid, Westley H Reeves, Laurence Morel

**Affiliations:** 1 Department of Medicine, Division of Rheumatology and Clinical Medicine, University of Florida, 1600 Archer Road, Gainesville, FL 32610-0275, USA; 2 Department of Pathology, Immunology, and Laboratory Medicine, University of Florida, 1600 Archer Road, Gainesville, FL 32610-0275, USA; 3 Department of Biostatistics, University of Florida, 1600 Archer Road, Gainesville, FL 32610-0275, USA; 4 Department of Medicine, Division of Rheumatology, Feinberg School of Medicine, Northwestern University, 240 East Huron Street, McGaw M360f, Chicago, IL 60611, USA; 5 School of Medicine, Emory University,101 Woodruff Circle, Woodruff Memorial Research Building, Suite 1315, Atlanta, GA 30322, USA

## Abstract

**Introduction:**

CD25^+ ^FOXP3^+ ^CD4^+ ^regulatory T cells (Tregs) are induced by transforming growth factor β (TGFβ) and further expanded by retinoic acid (RA). We have previously shown that this process was defective in T cells from lupus-prone mice expressing the novel isoform of the *Pbx1 *gene, *Pbx1-d*. This study tested the hypothesis that CD4^+ ^T cells from systemic lupus erythematosus (SLE) patients exhibited similar defects in Treg induction in response to TGFβ and RA, and that PBX1-d expression is associated with this defect.

**Methods:**

Peripheral blood mononuclear cells (PBMCs) were collected from 142 SLE patients and 83 healthy controls (HCs). The frequency of total, memory and naïve CD4^+ ^T cells was measured by flow cytometry on fresh cells. PBX1 isoform expression in purified CD4^+ ^T cells was determined by reverse transcription polymerase chain reaction (RT-PCR). PBMCs were stimulated for three days with anti-CD3 and anti-CD28 in the presence or absence of TGFβ and RA. The expression of CD25 and FOXP3 on CD4^+ ^T cells was then determined by flow cytometry. *In vitro *suppression assays were performed with sorted CD25^+ ^and CD25^- ^FOXP3^+ ^T cells. CD4^+ ^T cell subsets or their expansion were compared between patients and HCs with two-tailed Mann-Whitney tests and correlations between the frequencies of two subsets were tested with Spearman tests.

**Results:**

The percentage of CD25^- ^FOXP3^+ ^CD4^+ ^(CD25^- ^Tregs) T cells was greater in SLE patients than in HCs, but these cells, contrary to their matched CD25^+ ^counterparts, did not show a suppressive activity. RA-expansion of TGFβ-induced CD25^+ ^Tregs was significantly lower in SLE patients than in HCs, although SLE Tregs expanded significantly more than HCs in response to either RA or TGFβ alone. Defective responses were also observed for the SLE CD25^- ^Tregs and CD25^+ ^FOXP3^- ^activated CD4^+ ^T cells as compared to controls. PBX1-d expression did not affect Treg induction, but it significantly reduced the expansion of CD25^- ^Tregs and prevented the reduction of the activated CD25^+ ^FOXP3^- ^CD4^+ ^T cell subset by the combination of TGFβ and RA.

**Conclusions:**

We demonstrated that the induction of Tregs by TGFβ and RA was defective in SLE patients and that PBX1-d expression in CD4^+ ^T cells is associated with an impaired regulation of FOXP3 and CD25 by TGFβ and RA on these cells. These results suggest an impaired integration of the TGFβ and RA signals in SLE T cells and implicate the PBX1 gene in this process.

## Introduction

Systemic lupus erythematosus (SLE) is an autoimmune disease characterized by the production of pathogenic autoantibodies. Multiple studies have shown that these autoantibodies are T cell-dependent with autoreactive CD4^+ ^T cells providing co-stimulatory signals and cytokines such as IL-4 and IL-21 to the autoreactive B cells [1,2]. The CD4^+ ^T cells of SLE patients present many functional defects, which include a reduced number of circulating cells that is associated with disease activity [3-5], impaired signaling [6] and increased spontaneous activation coupled with a hypo-responsiveness upon reactivation [7,8].

The status of CD4^+ ^CD25^+ ^FOXP3^+ ^regulatory T cells (Tregs) in lupus has been examined by numerous studies. In the (NZB × NZW)F1 mouse model, Treg adoptive transfers delay and attenuate the course of disease [9]. In SLE patients, findings have been mixed [10-12]. Most studies have reported either decreased numbers of circulating Tregs that were inversely correlated with disease activity, or an abnormal suppressive activity. Other studies have, however, reported similar numbers or function of Tregs in SLE patients and healthy controls (HCs). A consensus has arisen that these discrepancies are most likely due to the lack of a rigorous definition of the markers used for Treg identification as well as to technical differences in Treg isolation. The CD4^+ ^CD25^- ^FOXP3^+ ^cell population (CD25^- ^Tregs) has been recently found to be expanded in SLE patients [13,14], but its origin and function are unclear [15]. One group working with newly diagnosed patients has suggested that CD25^- ^Tregs correspond to activated T cells without suppressive activity [13]. The other group working with treated patients has shown that the CD25^- ^Tregs retain a suppressive function, albeit incomplete, and have concluded that these cells represent an attempt to control active autoimmune activation [14].

The size of the Treg compartment results from the combined contribution of thymic-derived natural Tregs (nTregs) and peripherally induced Tregs (iTregs). Most of the studies in SLE patients have focused on circulating Tregs in which the relative contribution of nTregs and iTregs is unknown. Murine studies have shown that the TGFβ-dependent induction of iTregs is expanded by all-trans retinoic acid (RA) [16,17]. RA also expands the number of *de novo *TGFβ-induced human iTregs and enhances their suppressive activity [18]. Recent studies have now reported that RA also expands the number and enhances the function of murine [19] and human [20] nTregs. Therefore, RA stands out as a major regulator of the size and function of the Treg compartment.

We have reported that the murine *Sle1a.1 *lupus susceptibility locus results in the production of activated and autoreactive CD4^+ ^T cells, and in a reduction of the Treg pool [21,22]. In addition, *Sle1a.1 *CD4^+ ^T cells present a defective expansion of TGFβ-induced iTregs in response to RA (Cuda *et al*., in revision). At the molecular level, *Sle1a.1 *corresponds to an increased expression of a novel splice isoform of the pre-B cell leukemia homeobox 1 *Pbx1 *gene, *Pbx1-d*. PBX1 amino acid sequence and exon structure are entirely conserved between mouse and humans. We found that PBX1-d was expressed more frequently in the CD4^+ ^T cells from lupus patients than from HCs, and its presence in CD4^+ ^T cells correlated with an increased central memory population. The current study was designed to investigate whether *in vitro *induction of iTreg by TGFβ and RA was impaired in SLE patients as compared to HCs, and to determine whether PBX1-d expression played a role in the size of the Treg pool relative to TGFβ and RA exposure. We found that SLE patients with active renal disease have less Tregs than patients with inactive disease or HCs. We also confirmed that SLE patients carry more CD25^- ^FOXP3^+ ^CD4^+ ^(CD25^- ^Tregs) than HCs, and found that while the CD25^+ ^conventional Tregs showed variable levels of suppression, the CD25^- ^Tregs were uniformly non-suppressive (and, therefore, are not functionally speaking "Treg"). We found a defective regulation of CD25 and FOXP3 expression in response to TGFβ and RA in the CD4^+ ^T cells from SLE patients as compared to HCs, with SLE CD25^+ ^Tregs being more expanded by TGFβ and less by RA than HC CD25^+ ^Tregs. Interestingly, the combination of TGFβ and RA greatly expanded SLE activated CD25^+ ^FOXP3^- ^T cells as compared to HCs. PBX1-d expression was associated with greater numbers of CD25^- ^Tregs, but it significantly reduced their expansion by the combination of TGFβ and RA. Moreover, PBX1-d expression was associated with an impaired ability of TGFβ and RA to reduce the activated CD25^+ ^FOXP3^- ^CD4^+ ^T cell subset. Overall, we have demonstrated that the induction of Tregs by TGFβ and RA was defective in SLE patients and that PBX1-d expression in CD4^+ ^T cells impaired the regulation of FOXP3 and CD25 by TGFβ and RA on these cells. These results suggest an impaired integration of the TGFβ and RA signals in SLE T cells and implicate the PBX1 gene in this process.

## Materials and methods

### Study participants

Peripheral blood samples were obtained after signed informed consent in accordance with an IRB-reviewed protocol at the University of Florida. The diagnosis of SLE was established according to the 1982 revised American College of Rheumatology criteria. Disease activity was evaluated by the Systemic Lupus Erythematosus Disease Activity Index (SLEDAI) [23], a classic and validated measure [23]. At each visit, a urinalysis was obtained. For any patients showing abnormalities with hematuria or proteinuria, proteinuria was further quantitated by a spot microalbumin to creatinine (MAU/Cr) ratio [24]. In greater than 90% of the cases, renal involvement was confirmed by biopsy, and renal disease activity was defined as an MAU/Cr ratio greater than 500 mg/g. The SLE patients were then divided into three groups: inactive (SLEDAI <4), active non-renal (SLEDAI ≥4 and MAU/Cr ≤500), and active renal (SLEDAI ≥4; MAU/Cr >500). In the vast majority of the patients classified in the last group, renal disease dominated, with only relatively minor contributions from arthritis and skin manifestations, although organ non-specific blood work was also frequently abnormal. Patients with active non-renal disease presented skin and/or joint manifestations, and were overall less seriously ill than the patients with renal disease. The demographics of the patients and HCs are summarized in Table 1.

**Table 1:** Characteristics of human subjects used in this study

	Patients (142)		Controls (83)	
**Median age (range)**	**35 (20 to 74)**		**32 (19 to 61)**	
	**number**	**percentage**	**number**	**percentage**
Females	129	91%	55	66%
Males	13	9%	28	34%
Caucasians	62	43%	49	60%
African Americans	57	40%	18	22%
Hispanics	20	14%	2	2%
Asians	1	1%	8	10%
Mixed	4	3%	4	5%
PBX1-a	30	33%	28	56%
PBX1-a/d	25	27%	14	28%
PBX1-d	37	40%	8	!6%
**Medications**				
Steroids	62	42%		
No steroid	85	58%		
Mycophenolate mofetil	69	47%		
Methotrexate	7	5%		
Azathioprine	17	12%		
Cyclophosphamide	2	1%		
Abatacept	4	3%		
No immunosuppressive	47	32%		
Untreated	30	21%		
**Disease activity**				
Inactive	58	48%		
Active non-renal	14	12%		
Active renal	49	40%		

### T cell culture and flow cytometry

CD4^+ ^T cell subsets were analyzed by flow cytometry by staining with antibodies to CD3-PerCP (SP34-2; BD Biosciences, San Jose, CA, USA ), CD4-PC7 (SFCI12T4D11; Beckman Coulter, Brea, CA, USA), CD45RA-Pacific Blue (HI100; eBioscience, San Diego, CA, USA), CD45RO-F (UCHL1; BD Biosciences), CD62L-APC-AF70 (DREG56; eBioscience), FOXP3-APC (PCH101; eBioscience), or isotype controls. Anti-coagulated whole blood was incubated with the combination of antibodies at concentrations recommended by the manufacturer, subsequently lysed (BD FACS™; BD Biosciences) and fixed in 0.5% paraformaldehyde in PBS. In addition, gradient-purified (Ficoll; Sigma-Aldrich, St-Louis, MO, USA) PBMCs (5 × 10^5 ^cells/ml) were cultured for three days on plates coated with a combination of anti-CD3 (1 ug/ml), anti-CD28 (10 ug/ml) antibodies (BD Biosciences), and IL-2 (20 μg/m) in the presence or absence of 5 nM RA (Sigma-Aldrich) and TGFβ1 (Peprotech, Rocky Hill, NJ, USA). Cells were then stained with antibodies to CD3e (UCHT1; eBioscience), CD4-PC7 and CD25-PE (M-A251, BD Biosciences), followed by permeabilization (FOXP3 Fixation/Permeabilization Concentrate and Diluent; eBioscience) and staining for FOXP3-APC. Before using whole blood, the protocol was validated against isolated CD4^+ ^T cells, purified with RosetteSep (Stem Cell Technologies, Vancouver, BC, Canada) by negative selection, as previously described (Cuda *et al. *in revision). In a subset of samples, freshly harvested cells were also stained for CD3, CD4, CD127-PE (eBioscience) and CD25. The red blood cells (RBCs) were then lysed, the cells permeabilized and stained for FOXP3.

### T cell suppression assays

CD4^+ ^CD127^- ^T cells were enriched by negative selection from 6 ml of blood freshly collected in heparinized tubes following the manufacturer's instructions (RosetteSep Human CD4^+^CD127^low ^Regulatory T Cell Pre-Enrichment Cocktail; StemCell Technologies). A small aliquot was retained to verify purity (typically 70 to 80%), and the remaining cells were cultured for three days as described above for expansion of Tregs, using 20 ug TGFβ. After culture, the cells were harvested and stained under sterile conditions with a cocktail of anti-CD4-PE-Cy7, anti-CD25-Pacific Blue, and anti-CD127-PE. The cells were then suspended in PBS supplemented with 2% FBS and sorted with a FACSAria (BD Biosciences) into two populations (CD4^+ ^CD127^-^CD25^+ ^and CD4^+ ^CD127^- ^CD25^-^). An aliquot was retained for intracellular staining for FOXP3, as described above. The remaining purified CD25+ and CD25- Tregs were each resuspended in 500 ul of PBS, as were an aliquot of frozen PBMCs used as standardized responder cells, and an aliquot of standardized umbilical cord-derived Tregs, both prepared as previously described [25]. The responder cells were incubated with carboxyfluorescein succinimidyl ester (CFSE), while the Treg preparations were incubated with CellTrace Violet, both following the manufacturer's instructions (Invitrogen, Carlsbad, CA, USA). After quenching with FBS, 50,000 responder cells were added per well to a 96-well round-bottomed tissue culture plate pre-coated with anti-CD3 (2 μg/ml) and anti-CD28 (1 μg/ml) as previously described [25]. Tregs were added in triplicate at serial dilutions of 1:4 to 1:64. Additional controls included wells without Tregs (positive control) and wells without anti-CD3 and -CD28 stimulation (negative controls). Additional wells were prepared to which only Tregs were added. The cells were cultured for six days at 37°C, harvested, and stained with a combination of anti-CD3-PerCP, -CD4-PE-Cy7, and -CD8-APC. Cells were analyzed on a CyAn 9-color flow cytometer (Beckman Coulter). At least 2,500 events were collected in the lymphocyte gate and analyzed for CD8^+ ^T cell proliferation by FCS Express 4 RUO (DeNovo Software, Los Angeles, CA, USA). For evaluation of proliferation of Tregs, cells were gated for CD4 and excluded all CFSE^+ ^events. Control responder cells without Tregs showed that the CFSE^- ^and Cell Trace Violet populations did not merge. Proliferation indices, calculated as the ratios of the total gated cells at the end of culture over their initial number, and division indices, corresponding to CFSE dilution, were derived from the curve fitting data [26] and gave comparable results.

### PBX1 isoform analysis

Peripheral blood CD4^+ ^T cells were isolated from whole blood, as described above. The quality of isolation was verified by flow cytometry and was typically 80 to 90%. cDNA was synthesized from the purified CD4^+ ^T cells, and *Pbx1 *isoforms were detected with the following: 5' - GAA GTG CGG CAT CAC AGT CTC- 3' in exon 5, and 5' - ACT GTA CAT CTG ACT GGC TGC - 3' in exon 8.

### Statistical analysis

Statistical analyses were performed using GraphPad Prism 4. Data were presented as means ± SEM or scatter plots. Comparisons between two cohorts were performed with two-tailed Mann-Whitney tests and Dunns' multiple comparison tests when more than two groups were involved. Correlations were established using Spearman tests. Statistical significance obtained when *P *≤ 0.05 is indicated in the figures.

## Results

### Differential distribution of the memory and naïve CD4^+ ^T cell subsets between SLE patients and HCs

**Figure 1:**
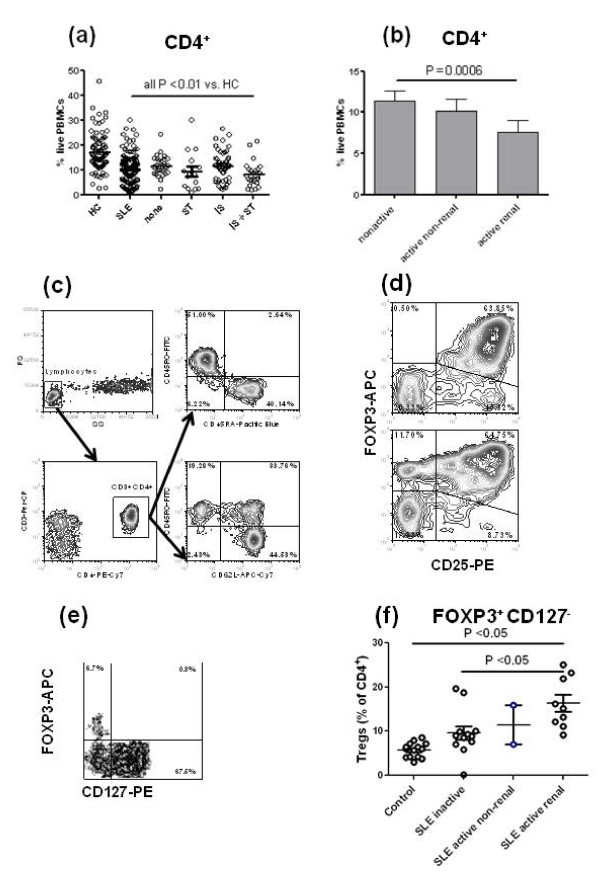
**CD3**^**+ **^**CD4**^**+ **^**T cell leucopenia in systemic lupus erythematosus (SLE) patients**. (**a**) Percentage of CD4^+ ^T cells in the peripheral blood mononuclear cells (PBMCs) of patients and healthy controls (HCs). CD4^+ ^T cell percentages was also compared between untreated patients (none, N = 28) and patients treated with either steroids alone (ST, N = 15) or immunosuppressive drugs alone (IS, N = 53) or both (IS + ST, N = 32). Each patient group was compared to HCs using Dunns' multiple comparison tests. (**b**) Percentage of CD4^+ ^T cells in the PBMCs of SLE patients according to their disease activity (non-active, active non-renal and active renal). (**c**) Representative PBMC fluorescence activated cell sorter (FACS) plots showing the CD45RO - CD45RA and CD45RO - CD62L stainings gated on CD3^+ ^CD4^+ ^lymphocytes. (**d**) Representative FACS plots showing FOXP3 and CD25 staining gated on CD4^+ ^lymphocytes of two PBMC samples three days after stimulation with anti-CD3 and anti-CD28. (**e**) Freshly obtained blood was stained with a combination of antibodies to CD3, CD4, CD25, and CD127. Following red blood cell lysis, the cells were permeabilized and stained for FOXP3 expression. The FACS plot shows a representative profile gated on CD3^+ ^CD4^+ ^lymphocytes, with the regulatory T cells (Tregs) being identified as FOXP3^+ ^CD127^-^. (**f**) Percentage of circulating Tregs identified as shown in (e) in HCs and SLE patients partitioned by disease activity.

The percentage of CD4^+ ^T cells was significantly lower in the PBMCs of SLE patients than in HCs (Figure 1a). All patients, either untreated or treated with steroids, or immunosuppressive drugs or both, presented a significantly lower percentage of CD4^+ ^T cells than HCs, indicating that treatment was not the main cause for low CD4^+ ^T cell counts. However, treatment was associated with a further decrease in the percentage of CD4^+ ^T cells (untreated patients: 11.51 ± 0.80%, patients treated with both steroids and immunosuppressive drugs: 8.06 ± 1.00%, *P *< 0.009). We also observed a significantly lower percentage of CD4^+ ^T cells in patients with active renal disease as compared to patients with inactive disease (Figure 1b). This difference associated with disease severity was not due to treatment as there was no difference between patients with inactive disease that were untreated or treated with either steroids or immunosuppressive drugs (12.44 ± 1.12%, N = 16 vs. 10.81 ± 0.88%, N = 50, respectively, *P *= 0.21). Finally, patients with inactive disease had a significantly lower percentage of CD4^+ ^T cells than in HCs (11.79 ± 0.80%, N = 52 vs. 17.12 ± 0.71%, N = 83, respectively, *P *< 0.0001). These results confirm earlier reports [3-5] that SLE patients present with CD4^+ ^T cell leucopenia correlated with disease activity and showed that it is accentuated by steroid and immunosuppressive treatment, which is by itself associated with disease activity.

**Figure 2:**
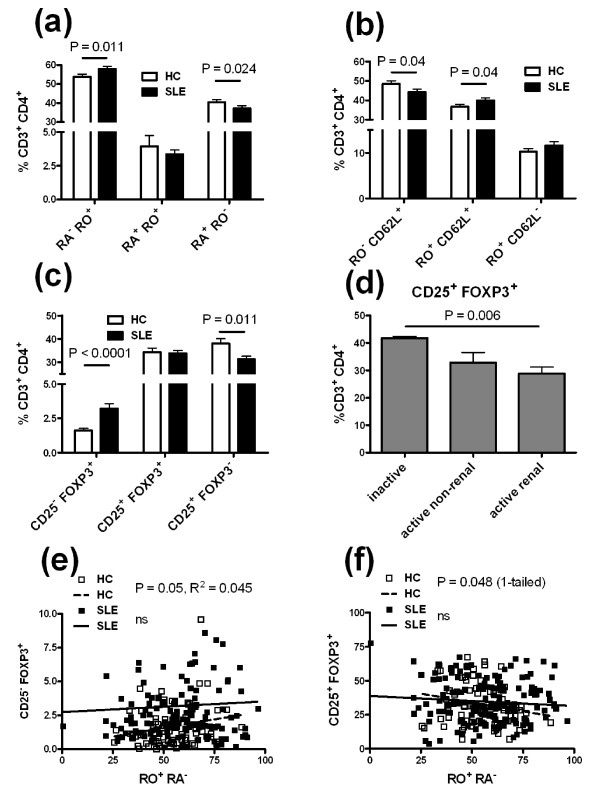
**Differential CD3**^**+ **^**CD4**^**+ **^**T cell subset distribution between healthy controls and systemic lupus erythematosus patients **Distribution of CD45RA^- ^CD45RO^+ ^(RA^- ^RO^+^) memory T cells and CD45RA^+ ^CD45RO^- ^(RA^+ ^RO^-^) naïve T cells (**a**), or CD45RO^- ^(RO^-^) CD62L^+ ^naïve T cells, CD45RO^+ ^(RO^+^) CD62L^+ ^central memory T cells and CD45RO^+ ^(RO^+^) CD62L^- ^effector memory T cells in the peripheral blood mononuclear cells (PBMCs) of SLE patients and HCs (**b**). (**c**) CD4^+ ^T cells activated for three days with anti-CD3 and anti-CD28 were compared between patients and HCs according to their CD25 and FOXP3 expression. (**d**) Percentage of expanded CD25^+ ^regulatory T cells (Tregs) in SLE patients according to their disease activity. (**e**) The percentage of CD25^- ^Tregs was positively correlated with the percentage of memory CD45RO^+ ^CD45RA^- ^CD4^+ ^T cells in HCs but not in patients. (**f**) The percentage of CD25^+ ^Tregs was negatively correlated (one-tail *P*-value) with the percentage of memory CD45RO^+ ^CD45RA^- ^CD4^+ ^T cells in HCs but not in patients. The graphs in (e-f) show the linear regression lines for HCs (dashed) and SLE patients (plain), the *P*-values for the Spearman correlation tests and the R^2 ^values calculated separately for the patient and HC cohorts. Ns, non-significant.

We compared the percentage of circulating CD45RA^+ ^CD45RO^- ^naïve and CD45RA^- ^CD45RO^+ ^memory CD4^+ ^T cells, and among the latter, the percentage of CD62L^+ ^CD45RO^+ ^central and CD62L^- ^CD45RO^+ ^effector memory T cells in the PBMCs of patients and HCs (Figure 1c). Patients presented significantly more memory T cells and less naïve CD4^+ ^T cells (identified as either CD45RA^+ ^CD45RO^- ^or CD62L^+ ^CD45RO^-^) than HCs (Figure 2a, b). Among memory T cells, it was the central but not the effector memory subset that was responsible for this difference (Figure 2b). Immunosuppressive treatment lowered the patients' memory/naïve CD4^+ ^T cell (*P *= 0.03) and the central memory/naïve T cell (*P *= 0.06) ratios. However, there was no difference between patients with active and inactive disease, or between patients that were treated or non-treated with steroids (data not shown).

### Differential distribution of expanded CD4^+ ^T cell subsets expressing CD25 and FOXP3 in SLE patients and HCs

FOXP3 and CD25 expression was quantified on CD4^+ ^T cells after three days of stimulation with anti-CD3 and anti-CD28 (Figure 1d). CD25^+ ^FOXP3^+ ^CD4^+ ^Tregs were present at similar levels in patients and HCs (Figure 2c). However, we found a significantly lower percentage of Tregs in patients with active renal disease than in patients with inactive disease, and patients with active non-renal disease presented an intermediate level (Figure 2d). As for the numbers of total CD4+ T cells, we believe that these results represent an association between decreased Treg levels and disease severity, rather than a tissue-specific association. Patients with active renal disease presented also significantly less Tregs than HCs (30.15 ± 1.75%, N = 58 vs. 35.46 ± 1.93% N = 78, respectively, *P *= 0.026). This indicated that the similar level of Tregs between SLE patients and HCs seen in Figure 2c was largely due to patients with inactive disease.

We also found a higher percentage of CD25^- ^Tregs in patients than in HCs, and conversely a lower percentage of CD25^+ ^FOXP3^- ^CD4^+ ^T cells in patients than in HCs (Figure 2c). The percentage of these two latter subsets did not vary with disease activity, or steroid or immunosuppressive treatment (data not shown). Because the amount of blood needed for all experiments was limiting, we did not use purified CD25^- ^CD4^+ ^T cells as the starting population. It is, therefore, possible that the reduced percentage of Tregs after culture merely resulted from a smaller starting population. However, we saw very few CD25^+ ^CD4^+ ^cells in freshly stained blood, indicating that selection for CD25^- ^T cells would have had little effect on our studies. More importantly, we also studied a subset of our freshly obtained samples for FOXP3 and CD4 co-expression. Because absence of CD127 has also been used as a marker of Tregs [27], this was also added to the staining strategy. As seen in Figure 1e, after gating on CD3^+ ^CD4^+ ^cells, the combination of FOXP3 and CD127 showed good separation of phenotypes, with the Tregs being identified as FOXP3^+ ^CD127^-^. A compilation of results showed that the starting population of Tregs was not decreased in our patient population compared to controls (Figure 1f). In fact, the active patients showed the highest starting levels, making it unlikely that our results with expanded T cells are due to a lower percentage of circulating Tregs.

We investigated whether there was a correlation between the level of CD45RA^- ^CD45RO^+ ^memory CD4^+ ^T cells and the size of the Treg subsets. The percentage of CD25^- ^Tregs was positively correlated with the percentage of memory T cells in HCs but not in patients (Figure 2e). There was a trend negatively correlating the percentage of CD25^+ ^Tregs cells with the percentage of memory T cells in HCs but not in patients (Figure 2f). Overall, these results show in HCs the expected positive correlation between CD25-Tregs and memory T cells and negative correlation between CD25^+ ^Tregs and memory T cells. The fact that these correlations were not observed for FOXP3^+ ^T cells in SLE patients suggests a defective homeostatic regulation of FOXP3 expression in SLE patients.

### SLE CD25^- ^Tregs do not suppress T cell proliferation

**Figure 3:**
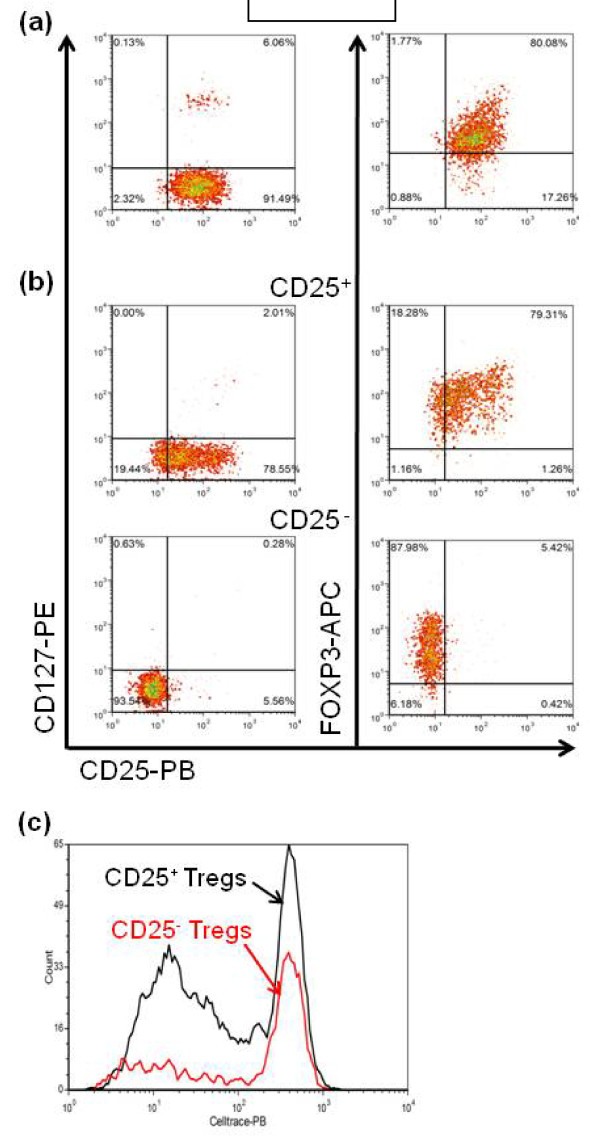
**Representative fluorescence activated cell sorter (FACS) plots showing the regulatory T cell (Treg) populations used in the suppression assays **(**a**) Standardized cord blood Treg used as positive controls, the great majority of which being CD127^- ^CD25^+ ^FOXP3^+^. (**b**) Treg isolated from a systemic lupus erythematosus **(**SLE) patient as CD4+ CD127-, then sorted as CD25^+ ^or CD25^- ^after stimulation and expansion with transforming growth factor beta (TGFβ) and retinoic acid (RA). The CD25^+^-sorted population was approximately 80% FoxP3^+ ^CD25^+^, while the CD25^-^-sorted population was more than 80% FoxP3^+ ^CD25^-^. (**c**) Proliferation of CD25^+ ^and CD25^- ^Treg isolated from a same patient in the presence of standardized peripheral blood mononuclear cells (PBMCs) at the same dilution (1:4), in the presence of anti-CD3 and anti-CD28 for six days, showing a robust response of the CD25^+ ^as opposed to the CD25^- ^Tregs.

**Figure 4:**
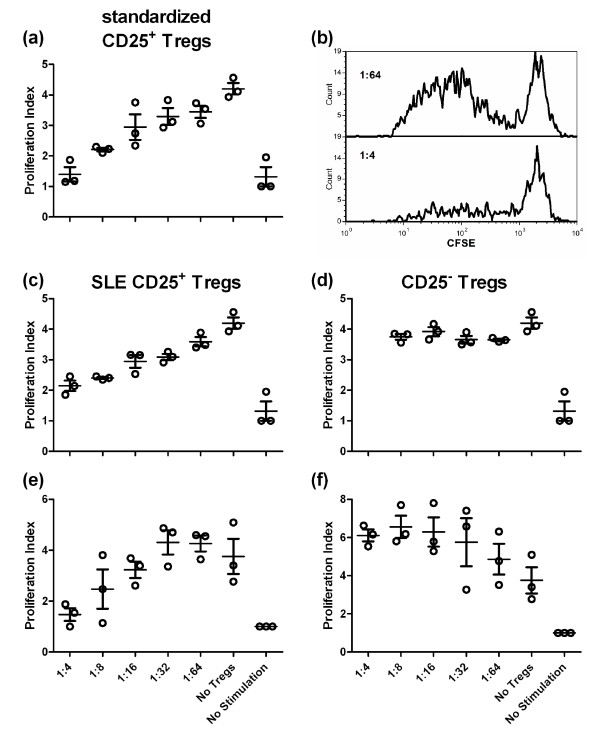
**CD25**^**+ **^**but not CD25**^- ^**regulatory T cells (Tregs) expanded from systemic lupus erythematosus (SLE) patients suppressed T cell proliferation**. Standardized aliquots of peripheral blood mononuclear cells (PBMCs) were cultured for six days in the presence of standardized Tregs (**a**), CD25^+ ^(**c**, **e**) or CD25^- ^(**d**, **f**) Tregs expanded *in vitro *from the PBMCs of SLE patients in the presence of transforming growth factor beta (TGFβ) and retinoic acid (RA). (c-d) and (e-f) CD25^+ ^and CD25^- ^Tregs were obtained from a same patient. Representative profiles of the CD8^+ ^PBMC proliferation in the presence of CD25^+ ^Tregs at the indicated dilutions are depicted (**b**). A varying amount of suppression was mediated by the CD25+ population, while the CD25^- ^population showed either no effect (top) or appeared to promote proliferation (bottom). These data are representative of six patients prepared in three independent experiments.

The function of the CD25^- ^FOXP3^+ ^CD4^+ ^T cells that is expanded in SLE patients is controversial [13,14]. We, therefore, assessed the suppressive capacity of these cells comparatively to their CD25^+ ^FOXP3^+ ^CD4^+ ^counterparts in our SLE cohort. As a positive control, we used standardized Treg isolated from cord blood, which were, as expected, largely CD127^- ^FOXP3^+ ^CD25^+ ^cells (Figure 3a). CD4^+ ^CD127^- ^cells isolated from patients' PBMCs were expanded by stimulation with anti-CD3 and CD28, TGFβ and RA, then sorted into CD25^+ ^and CD25^- ^populations. As shown in Figure 3b, this protocol led to a good separation of CD127^- ^FOXP3^+ ^CD25^+ ^and CD127^- ^FOXP3^+ ^CD25^- ^populations. These cells were then used in standard T cell suppression assays. As expected, the cord blood standardized Tregs showed a robust suppression (Figure 4a). CD25^+ ^Tregs from lupus patients also showed strong suppression (Figure 4b, c, e), although to a lesser extent in some patients (data not shown), which is consistent with reports of altered Treg function in some SLE patients [28]. To the contrary, none of the CD25^- ^Tregs isolated from six different patients showed any suppressive activity (Figure 4d, f). In one patient, the CD25^- ^Tregs actually stimulated the CD8^+ ^allogeneic T cells (Figure 4f). Furthermore, contrary to the CD25^+ ^Tregs, the CD25^- ^Tregs proliferated poorly in the stimulated co-cultures with PBMCs (Figure 3c). While the data depicted reflect proliferation of the CD8^+ ^PBMCs, comparable results were obtained for CD4+ PBMCs, although proliferation was less robust (data not shown). These results show that the CD25^- ^Tregs isolated from our cohort of SLE patients have lost their suppressive function.

### Differential response of CD4^+ ^T cells to TGFβ and retinoic acid in SLE patients and HCs

**Figure 5:**
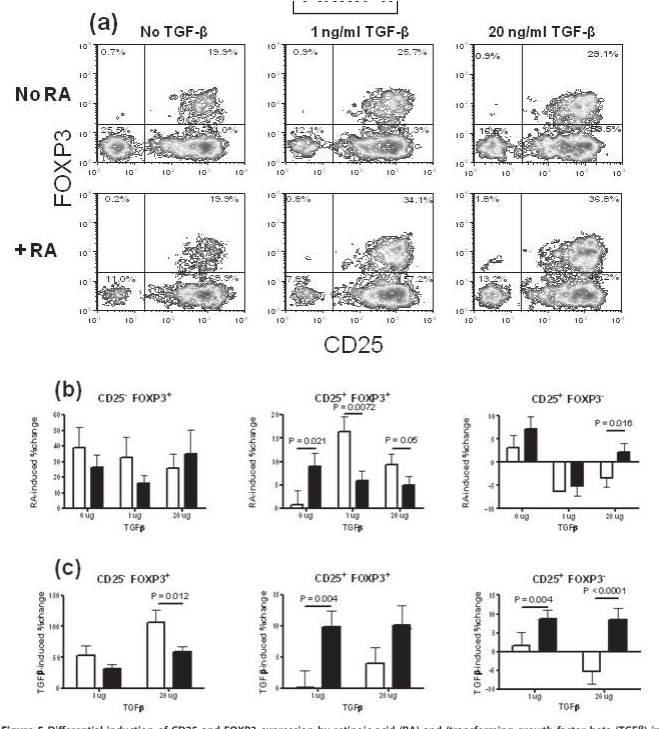
**Differential induction of CD25 and FOXP3 expression by retinoic acid (RA) and (transforming growth factor beta (TGFβ) in healthy controls (HCs) and systemic lupus erythematosus (SLE) patients**. (**a**) Representative fluorescence activated cell sorter (FACS) plots showing FOXP3 and CD25 staining in CD4^+ ^gated peripheral blood mononuclear cells (PBMCs) after three days stimulation with anti-CD3 and anti-CD28 with or without RA and in the presence of 0, 1, or 20 ug/ml of TGFβ. In the (b-d) panels, CD25^- ^regulatory T cells (Tregs) are shown on the left, Tregs in the middle, and CD25^+ ^FOXP3^- ^CD4^+ ^T cells on the right. (**b**) RA-induced expansion in the presence of 0, 1, or 20 ug/ml of TGFβ. The graphs show the ((RA - no RA)/no RA) values for each TGFβ concentration. (**c**) TGFβ-induced expansion in the absence of RA. The graphs show the ((TGFβ - no TGFβ)/no TGFβ) values for each concentration of TGFβ. HCs are represented by white symbols and SLE patients by black symbols.

We systematically compared the effect of TGFβ and RA on CD25 and FOXP3 expression by CD4^+ ^T cells from SLE patients and HCs stimulated with anti-CD3 and anti-CD28 (Figure 5a). As shown in Figure 5b, RA expanded CD25^- ^Tregs to a similar level between HCs and patients. The effect of RA on CD25^+ ^Treg expansion depended on the presence of TGFβ: In the absence of TGFβ, CD25^+ ^Tregs were expanded by RA significantly more in patients than in HCs. In the presence of either 1 or 20 ug/ml of TGFβ, the opposite result was observed, that is, RA expanded Tregs less in patients than in HCs. CD25^+ ^FOXP3^- ^CD4^+ ^T cells were expanded by RA alone to a similar level in HCs and patients. In the presence of 1 ug/ml of TGFβ, the percentage of CD25^+ ^FOXP3^- ^CD4^+ ^T cells was decreased by RA to a similar extent between HCs and patients. When the concentration of TGFβ reached 20 ug/ml, RA still decreased the percentage of CD25^+ ^FOXP3^- ^CD4^+ ^T cells in HCs but increased it in SLE patients, leading to a significant difference between the two cohorts.

In the absence of RA, TGFβ alone expanded the CD4^+ ^T cell subsets differently between HCs and SLE patients (Figure 5c). CD25^- ^Tregs were expanded significantly less in SLE patients than in HCs by 20 ug/ml TGFβ. To the contrary, TGFβ expanded CD25^+ ^Tregs more in patients than in HCs, and the difference was highly significant with 1 ug/ml TGFβ (*P *< 0.01). TGFβ also expanded CD25^+ ^FOXP3^- ^CD4^+ ^T cells significantly more in patients than in HCs at both concentrations. Interestingly, 20 ug/ml of TGFβ expanded CD25^+ ^FOXP3^- ^CD4^+ ^T cells in patients while it shrunk this subset in HCs, as previously noted for RA in the presence of the same amount of TGFβ (Figure 5b). Overall, these results revealed a differential response of the CD4^+ ^T cell subsets to TGFβ and RA between SLE patients and HCs.

### Memory CD4^+ ^T cells are associated with a lower Treg induction in SLE patients

**Figure 6:**
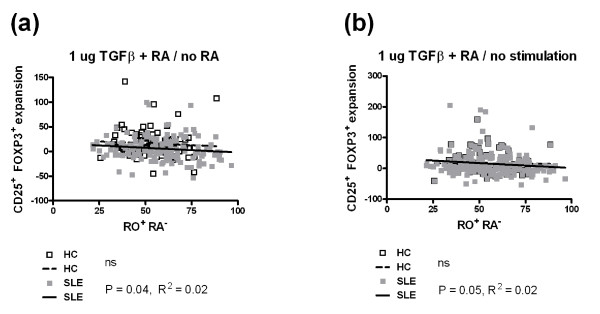
**Memory CD45RO**^**+ **^**CD45RA**^- ^**CD4**^**+ **^**T cells are associated with a lower induction of FOXP3 by (transforming growth factor beta) TGFβ and retinoic acid (RA) in systemic lupus erythematosus (SLE) patients**. (**a**) Treg expansion by RA in the presence of 1 ug/ml of TGFβ was negatively correlated with the percentage of memory CD4^+ ^T cells in the peripheral blood mononuclear cells (PBMCs) of SLE patients but not healthy controls (HCs). (**b**) Treg expansion by the combination of RA and 1 ug/ml of TGFβ over the absence of both RA and TGFβ was negatively correlated with the percentage of memory CD4^+ ^T cells in the PBMCs of SLE patients but not HCs. HCs are represented by white symbols and dashed linear regression lines; SLE patients are represented by grey symbols and plain linear regression lines. The *P*-values for Spearman correlation tests and the R^2 ^values are shown for the patient and HC cohorts. Ns, non-significant.

Memory CD4^+ ^T cells interfere with the TGFβ and RA-mediated conversion of naïve T cells into Tregs in both mice [29] and humans [18]. We investigated whether this occurred in our experimental conditions and whether differences existed between SLE patients and HCs. We evaluated correlations between the expansion of the CD25 FOXP3 subsets with the percentage of either total CD45RO^+ ^CD45RA^- ^memory CD4^+ ^T cells, CD45RO^+ ^CD62L^+ ^central memory or CD45RO^+ ^CD62L^- ^effector memory T cells. Similar results were obtained for total memory (Figure 6) and central memory CD4^+ ^T cells (data not shown), but the significance was always higher for the total memory CD4^+ ^T cells. We also investigated these correlations in the six combinations of RA and TGFβ used in this study (Figure 5a), and we show only the most representative combinations that showed significant results.

The expansion of Tregs by RA in the presence of 1 ug/ml of TGFβ (Figure 6a) or by the combination of RA and 1 ug/ml of TGFβ (Figure 6b) was negatively correlated with the percentage of memory CD4^+ ^T cells in the PBMCs of SLE patients. There was a trend in the same direction for HCs, and the negative correlations were highly significant for the combined cohorts (data not shown). No correlation was observed between the expansion of CD25^- ^Tregs by the combination of RA and 20 ug/ml of TGFβ and the percentage of memory CD4^+ ^T cells in the PBMCs of either SLE patients or HCs (data not shown).. Finally, the expansion of CD25^+ ^FOXP3^- ^CD4^+ ^T cells by any combination of RA and TGFβ was not correlated with the percentage of memory CD4^+ ^T cells in either SLE patients or HCs (data not shown). Overall, these results suggest that the presence of memory T cells interferes with the expansion of Tregs by RA and TGFβ more in SLE patients than in HCs, possibility because of the higher frequency of the memory T cells in patients.

### The expansion of the CD25 FOXP3 CD4^+ ^subsets by RA and TGFβ is affected by expression of the PBX1-d isoform

*Pbx1-d *over-expression is associated with an increased CD4^+ ^T cell activation and a reduced Treg number and function. Furthermore, we have shown that murine CD4^+ ^T cells expressing *Pbx1-d *and human Jurkat T cells transfected with *PBX1-d *presented a defective response to RA (Cuda *et al.*, in revision). *PBX1-d *was also expressed significantly more frequently in the CD4^+ ^T cells from SLE patients than from HCs (Cuda *et al.*, in revision). In the entire cohort combining SLE patients and HCs, PBX1-d expression was associated with CD4^+ ^T cell leucopenia and higher ratios of memory to naïve CD4^+ ^T cells. These results prompted us to examine whether the expansion of the CD25 FOXP3 CD4^+ ^subsets by RA and TGFβ were affected by the expression of the *PBX1 *isoforms.

**Figure 7:**
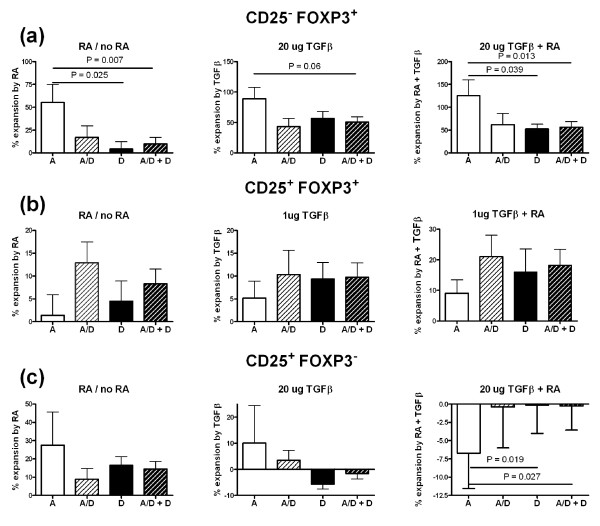
**PBX1-D expression affects FOXP3 and CD25 induction by retinoic acid and (transforming growth factor beta)**. Combined CD4^+ ^T cells from systemic lupus erythematosus patients (SLE) and healthy controls (HCs) were partitioned according to their expression of the PBX1-A (white), PBX1-D (black), co-expression of both PBX1-A and PBX1-D (A/D, light hatched) or either PBX1-D or PBX1-A/D (heavy hatched) isoforms. The expansion of CD25^- ^regulatory T cells (Tregs) (**a**), Tregs (**b**) and CD25^+ ^FOXP3^- ^CD4^+ ^T cells **(c) **is shown by retinoic acid (RA) alone (left panels), transforming growth factor beta (TGFβ) alone (middle panels), or the combination of the two (right panels).

The expression of the PBX1-d isoform was associated with a significantly decreased expansion of CD25^- ^Tregs by RA alone or by the combination of RA and TGFβ (Figure 7a). The same trend was observed for their expansion by TGFβ alone. The percentage of CD25^- ^Tregs found prior to RA and TGFβ expansion was higher in the SLE patients than in HCs (Figure 2c). When the samples were partitioned according to the PBX1 isoform, individuals expressing the PBX1-d isoform presented significantly higher levels of CD25^- ^Tregs prior to RA and TGFβ expansion than individuals with only the PBX1-a isoform (3.38 ± 0.37% vs. 2.30 ± 0.34%, respectively, *P *= 0.0035). These results indicate that PBX1-d is associated with a higher level of CD25^- ^Tregs, but to a decreased expansion of these cells in response to RA or TGFβ. The PBX1 isoforms did not affect the expansion of Tregs by RA, TGFβ, or the combination of the two (Figure 7b). The same result was obtained with all the combinations of RA and TGFβ tested in this study (data not shown). Finally, the expansion of CD25^+ ^FOXP3^- ^CD4^+ ^T cells by either RA or TGFβ alone was not affected by PBX1 isoform expression (Figure 7c, left and center). However, the combination of RA or TGFβ reduced the percentage of CD25^+ ^FOXP3^- ^CD4^+ ^T only when these cells expressed PBX1-a, while the percentage of cells expressing PBX1-d was not changed by RA and TGFβ (Figure 5c, right). Overall these results suggest that PBX1-d expression is involved in FOXP3 and CD25 expression, and that it may interfere with RA and TGFβ signals in CD4^+ ^T cells.

## Discussion

Many studies have examined the number and function of Tregs in lupus patients, but to our knowledge none has examined the ability of iTregs from lupus patients to be induced and expanded *in vitro *as compared to HCs. Basic parameters of our lupus cohort confirmed previous findings, such as reduced numbers of CD4^+ ^T cells, skewed memory to naïve CD4^+ ^T cell ratios and a reduced CD25^+ ^Treg compartment in patients with active renal disease. Mouse studies have shown a functional link between lymphopenia and Treg instability [30], and suggest that the CD4^+ ^T cell lymphopenia and reduced Treg numbers found in SLE patients may also be linked. We also confirmed the expansion of CD25^- ^Tregs in SLE patients, indicating that our study population was similar to most cohorts that have been recently used in the field. *In vitro *assays showed, however, that this FOXP3^+ ^subset is not suppressive, while the matched CD25^+ ^FOXP3^+ ^cells were suppressive to variable, but significant levels. These data support Yang *et al.*'s findings [13], indicating that the CD25^- ^FOXP3^+ ^population that is expanded in SLE patients corresponds to either activated T cells or to "ex-Treg" that have lost their suppressive activity. Further analyses, including the methylation status of the FOXP3 locus, will be necessary to distinguish these possibilities.

A defective homeostatic regulation of FOXP3 expression in SLE CD4^+ ^T cells was indicated by the absence of the correlations found in HCs. The inverse correlation between memory T cells and Tregs reported in our HC cohort corresponds to the reciprocal balance between immune suppression and inflammation [31]. The positive correlation that we observed between the percentage of CD25^- ^Tregs and memory T cells in HCs could be interpreted either as these cells representing an activated non-regulatory subset [13] or a response to high levels of activation [14]. Nonetheless, these correlations between FOXP3 expressing T cells and memory T cells did not exist in lupus patients, while a positive correlation between CD25^+ ^FOXP3^- ^CD4^+ ^T cells and memory T cells was maintained. This strongly suggests a defect in homeostatic regulation of FOXP3^+ ^T cells in lupus patients. A recent study has shown that the mechanisms involved in balancing Th1 and Th17 regulation are defective in lupus patients [32]. Given the plasticity of the CD4^+ ^T cell subsets [33], future studies should determine whether the defective regulations of FOXP3^+ ^T cells and Th1/Th17 T cells in lupus patients are functionally related.

As expected, RA expanded the TGFβ induction of Tregs and decreased the proportion of CD25^+ ^FOXP3^- ^CD4^+ ^T cells in HCs. The effects of RA and TGFβ on SLE T cells were, however, more complex, and different when considered singly or in combination. TGFβ alone expanded significantly more lupus Tregs than HC Tregs. Several studies have found decreased levels of TGFβ in SLE patients [34] (although a recent one did not find any difference [32]), and the enhanced response that we observed in SLE Tregs may represent a consequence of a relative *in vivo *TGFβ starvation. RA alone also expanded SLE Tregs, while there was no expansion of HC Tregs. In the presence of TGFβ, however, the benefit of RA exposure was significantly less for SLE Treg expansion than for HC Treg expansion. This suggests that the integration of the TGFβ and RA signals might be defective in lupus T cells, which will have to be investigated systematically at the cellular and molecular levels. Several mechanisms have been proposed for RA expansion of TGBβ-induced Tregs, including by enhancing Foxp3 transcription and counteracting IL-6 signaling [19,35], or blocking CD4^+ ^CD44^hi ^memory cells from inhibiting iTreg differentiation [29]. The negative correlation that we have found between the levels of memory T cells and Treg expansion by the combination of TGFβ and RA in SLE patients suggests that at least the latter mechanism is defective, either because there are too many memory T cells or they are refractory to RA inhibition. Interestingly, the CD25^- ^Tregs were also expanded by the combination of RA and TGBβ, and these cells responded less to TGBβ and to the combination of TGBβ and RA in SLE patients than HCs. Finally, the CD25^+ ^FOXP3^- ^CD4^+ ^T cells responded to TGBβ and RA in opposite directions between SLE patients and HC controls, with an expansion in the former and a reduction in the latter. Overall, these results suggest that the integration of the TGBβ and RA pathways that are involved in the induction of CD4^+ ^T cell subsets are defective in lupus patients. A pro-inflammatory role of RA has been recently discovered when it is expressed with high levels of IL-15 in the gut [36]. SLE patients express high levels of pro-inflammatory cytokines; therefore, creating a milieu that may promote RA pro-inflammatory role, a hypothesis that will have to be tested in future studies.

*Pbx1 *is a transcription factor whose function is linked to RA [37]. Its role in adult immune cells has not yet been described, except for macrophages in which it regulates the production of IL-10 in response to apoptotic cells [38]. By positional cloning, we have determined that *Pbx1 *regulates the production of autoreactive CD4^+ ^T cells and the size of the Treg compartment in the NZM2410 lupus model. Pbx1-d also altered the responses of CD4^+ ^T cells to RA. Based on the homology between murine and human *PBX1*, we investigated the expression of PBX1-d, the isoform over-expressed in the NZM210 allele, in lupus patients' CD4^+ ^T cells. We found not only that PBX1-d was over expressed in lupus patients as compared to normal controls, but that PBX1-d expression in the general population was associated with decreased CD4^+ ^T cell numbers and increased levels of memory CD4^+ ^T cells (Cuda *et al.*, in revision). In this study, we found that PBX1-d expression had no effect of CD25^+ ^Treg expansion by TGFβ and RA. PBX1-d was however associated with a higher level of CD25^- ^Tregs, but to a defective expansion of these cells in response to TGFβ and RA. The nature of the molecular events by which PBX1-d promotes CD25^- ^Treg expansion remains to be determined.

## Conclusions

Overall, the expression of the PBX1-d isoform that is significantly associated with SLE in both murine and human T cells impacts the homeostasis of memory T cells (Cuda *et al.*, in revision) and regulatory T cells, including that of CD25^- ^Tregs that we have found to have lost their regulatory functions (this study). This represents a novel mechanism of auto-reactive T cell regulation that needs to be elucidated at the molecular level.

## Abbreviations

CFSE: carboxyfluorescein succinimidyl ester; HCs: healthy controls; iTregs: induced Tregs; nTreg: natural Tregs; PBMCs: peripheral blood mononuclear cells; RA: all trans retinoic acid; SLE: systemic lupus erythematosus; SLEDAI: Systemic Lupus Erythematosus Disease Activity Index; Tregs: CD4^+ ^CD25^+ ^FOXP3^+ ^regulatory T cells.

## Competing interests

The authors declare that they have no competing interests.

## Authors' contributions

ES and LM had full access to all of the data in the study and took responsibility for the integrity of the data as well as for the preparation of the manuscript. They designed the study and analyzed the data. TB participated in the design of the suppression assays and provided reagents. ES and WR recruited the patients. EB, AA and SW performed the experiments. WH supervised the statistical analysis. CC participated in the study design. All authors have read and approved the manuscript.

## References

[B1] Shlomchik MJ Craft JE Mamula MJ From T to B and back again: positive feedback in systemic autoimmune disease Nat Rev Immunol 2001 1 147 153 10.1038/35100573 11905822 11905822

[B2] La Cava A Lupus and T cells Lupus 2009 18 196 201 10.1177/0961203308098191 19213856

[B3] Messner RP Lindstrom FD Williams RC Jr Peripheral blood lymphocyte cell surface markers during the course of systemic lupus erythematosus J Clin Invest 1973 52 3046 3056 10.1172/JCI107503 4584345 PMC302579

[B4] Glinski W Gershwin ME Steinberg AD Fractionation of cells on a discontinuous Ficoll gradient. Study of subpopulations of human T cells using anti-T-cell antibodies from patients with systemic lupus erythematosus J Clin Invest 1976 57 604 614 10.1172/JCI108316 55418 PMC436693

[B5] Rivero SJ az-Jouanen E Alarcon-Segovia D Lymphopenia in systemic lupus erythematosus. Clinical, diagnostic, and prognostic significance Arthritis Rheum 1978 21 295 305 10.1002/art.1780210302 646828

[B6] Tsokos GC Nambiar MP Tenbrock K Juang YT Rewiring the T-cell: signaling defects and novel prospects for the treatment of SLE Trends Immunol 2003 24 259 263 10.1016/S1471-4906(03)00100-5 12738420

[B7] Murashima A Takasaki Y Ohgaki M Hashimoto H Shirai T Hirose S Activated peripheral blood mononuclear cells detected by murine monoclonal antibodies to proliferating cell nuclear antigen in active lupus patients J Clin Immunol 1990 10 28 37 10.1007/BF00917495 1968905

[B8] Dawisha SM Gmelig-Meyling F Steinberg AD Assessment of clinical parameters associated with increased frequency of mutant T cells in patients with systemic lupus erythematosus Arthritis Rheum 1994 37 270 277 10.1002/art.1780370217 8129782

[B9] Scalapino KJ Tang Q Bluestone JA Bonyhadi ML Daikh DI Suppression of disease in New Zealand Black/New Zealand White lupus-prone mice by adoptive transfer of *ex vivo *expanded regulatory T cells J Immunol 2006 177 1451 1459 16849451 10.4049/jimmunol.177.3.1451

[B10] Horwitz DA Regulatory T cells in systemic lupus erythematosus: past, present and future Arthritis Res Ther 2008 10 227 10.1186/ar2511 19040771 PMC2656253

[B11] Bonelli M Smolen JS Scheinecker C Treg and lupus Ann Rheum Dis 2010 69 i65 i66 10.1136/ard.2009.117135 19995748

[B12] La Cava A The busy life of regulatory T cells in systemic lupus erythematosus Discov Med 2009 8 13 17 19772836

[B13] Yang HX Zhang W Zhao LD Li Y Zhang FC Tang FL He W Zhang X Are CD4+CD25-Foxp3+ cells in untreated new-onset lupus patients regulatory T cells? Arthritis Res Ther 2009 11 R153 10.1186/ar2829 19821980 PMC2787292

[B14] Bonelli M Savitskaya A Steiner CW Rath E Smolen JS Scheinecker C Phenotypic and functional analysis of CD4+CD25-Foxp3+ T cells in patients with systemic lupus erythematosus J Immunol 2009 182 1689 1695 19155519 10.4049/jimmunol.182.3.1689

[B15] Horwitz D Identity of mysterious CD4+CD25-Foxp3+ cells in SLE Arthritis Res Ther 2010 12 101 10.1186/ar2894 20122288 PMC2875622

[B16] Benson MJ Pino-Lagos K Rosemblatt M Noelle RJ All-trans retinoic acid mediates enhanced T reg cell growth, differentiation, and gut homing in the face of high levels of co-stimulation J Exp Med 2007 204 1765 1774 10.1084/jem.20070719 17620363 PMC2118687

[B17] Bettelli E Carrier Y Gao W Korn T Strom TB Oukka M Weiner HL Kuchroo VK Reciprocal developmental pathways for the generation of pathogenic effector TH17 and regulatory T cells Nature 2006 441 235 238 10.1038/nature04753 16648838

[B18] Wang J Huizinga TW Toes RE De novo generation and enhanced suppression of human CD4+CD25+ regulatory T cells by retinoic acid J Immunol 2009 183 4119 4126 10.4049/jimmunol.0901065 19717521

[B19] Zhou X Kong N Wang J Fan H Zou H Horwitz D Brand D Liu Z Zheng SG Cutting Edge: All-trans retinoic acid sustains the stability and function of natural regulatory T cells in an inflammatory milieu J Immunol 2010 185 2675 2679 10.4049/jimmunol.1000598 20679534 PMC3098624

[B20] Golovina TN Mikheeva T Brusko TM Blazar BR Bluestone JA Riley JL Retinoic acid and rapamycin differentially affect and synergistically promote the *ex vivo *expansion of natural human T regulatory cells PLoS ONE 2011 6 e15868 10.1371/journal.pone.0015868 21253593 PMC3017077

[B21] Chen Y Cuda C Morel L Genetic determination of T cell help in loss of tolerance to nuclear antigens J Immunol 2005 174 7692 7702 15944270 10.4049/jimmunol.174.12.7692

[B22] Cuda CM Zeumer L Sobel ES Croker BP Morel L Murine lupus susceptibility locus Sle1a requires the expression of two sub-loci to induce inflammatory T cells Genes Immun 2010 11 542 553 10.1038/gene.2010.23 20445563 PMC2958247

[B23] Griffiths B Mosca M Gordon C Assessment of patients with systemic lupus erythematosus and the use of lupus disease activity indices Best Pract Res Clin Rheumatol 2005 19 685 708 10.1016/j.berh.2005.03.010 16150398

[B24] Guy M Borzomato JK Newall RG Kalra PA Price CP Protein and albumin-to-creatinine ratios in random urines accurately predict 24 h protein and albumin loss in patients with kidney disease Ann Clin Biochem 2009 46 468 476 10.1258/acb.2009.009001 19729498

[B25] Putnam AL Brusko TM Lee MR Liu W Szot GL Ghosh T Atkinson MA Bluestone JA Expansion of human regulatory T-cells from patients with type 1 diabetes Diabetes 2009 58 652 662 19074986 10.2337/db08-1168 PMC2646064

[B26] Roederer M Interpretation of cellular proliferation data: avoid the panglossian Cytometry A 2011 79 95 101 21265003 10.1002/cyto.a.21010

[B27] Liu W Putnam AL Xu-Yu Z Szot GL Lee MR Zhu S Gottlieb PA Kapranov P Gingeras TR Fazekas de St Groth B Clayberger C Soper DM Ziegler SF Bluestone JA CD127 expression inversely correlates with FoxP3 and suppressive function of human CD4+ T reg cells J Exp Med 2006 203 1701 1711 10.1084/jem.20060772 16818678 PMC2118339

[B28] Scheinecker C Bonelli M Smolen JS Pathogenetic aspects of systemic lupus erythematosus with an emphasis on regulatory T cells J Autoimmun 2010 35 269 275 10.1016/j.jaut.2010.06.018 20638240

[B29] Hill JA Hall JA Sun CM Cai Q Ghyselinck N Chambon P Belkaid Y Mathis D Benoist C Retinoic acid enhances Foxp3 induction indirectly by relieving inhibition from CD4 + CD44hi cells Immunity 2008 29 758 770 10.1016/j.immuni.2008.09.018 19006694 PMC3140207

[B30] Zhou X Bailey-Bucktrout S Jeker LT Bluestone JA Plasticity of CD4(+) FoxP3(+) T cells Curr Opin Immunol 2009 21 281 285 10.1016/j.coi.2009.05.007 19500966 PMC2733784

[B31] Sakaguchi S Ono M Setoguchi R Yagi H Hori S Fehervari Z Shimizu J Takahashi T Nomura T Foxp3+ CD25+ CD4+ natural regulatory T cells in dominant self-tolerance and autoimmune disease Immunol Rev 2006 212 8 27 10.1111/j.0105-2896.2006.00427.x 16903903

[B32] Shah K Lee WW Lee SH Kim SH Kang SW Craft J Kang I Dysregulated balance of Th17 and Th1 cells in systemic lupus erythematosus Arthritis Res Ther 2010 12 R53 10.1186/ar2964 20334681 PMC2888202

[B33] Murphy KM Stockinger B Effector T cell plasticity: flexibility in the face of changing circumstances Nat Immunol 2010 11 674 680 20644573 10.1038/ni.1899 PMC3249647

[B34] Barreto M Ferreira R Lourenco L Moraes-Fontes M Santos E Alves M Carvalho C Martins B Andreia R Viana J Vasconcelos C Mota-Vieira L Ferreira C Demengeot J Vicente A Low frequency of CD4+CD25+ Treg in SLE patients: a heritable trait associated with CTLA4 and TGFb gene variants BMC Immunol 2009 10 5 10.1186/1471-2172-10-5 19173720 PMC2656467

[B35] Xiao S Jin H Korn T Liu SM Oukka M Lim B Kuchroo VK Retinoic acid increases Foxp3+ regulatory T cells and inhibits development of Th17 cells by enhancing TGF-beta-driven Smad3 signaling and inhibiting IL-6 and IL-23 receptor expression J Immunol 2008 181 2277 2284 18684916 10.4049/jimmunol.181.4.2277 PMC2722959

[B36] DePaolo RW Abadie V Tang F Fehlner-Peach H Hall JA Wang W Marietta EV Kasarda DD Waldmann TA Murray JA Semrad C Kupfer SS Belkaid Y Guandalini S Jabri B Co-adjuvant effects of retinoic acid and IL-15 induce inflammatory immunity to dietary antigens Nature 2011 471 220 224 10.1038/nature09849 21307853 PMC3076739

[B37] Qin P Haberbusch JM Zhang Z Soprano KJ Soprano DR Pre-B cell leukemia transcription factor (PBX) proteins are important mediators for retinoic acid-dependent endodermal and neuronal differentiation of mouse embryonal carcinoma P19 cells J Biol Chem 2004 279 16263 16271 10.1074/jbc.M313938200 14742427

[B38] Chung EY Liu J Homma Y Zhang Y Brendolan A Saggese M Han J Silverstein R Selleri L Ma X Interleukin-10 expression in macrophages during phagocytosis of apoptotic cells is mediated by homeodomain proteins Pbx1 and Prep-1 Immunity 2007 27 952 964 10.1016/j.immuni.2007.11.014 18093541 PMC2194654

